# Supplementary material for: AI literacy among basic school teachers in Ghana: A structural equation modelling analysis
Source: PLoS One. 2026 Apr 17;21(4):e0347117. doi: 10.1371/journal.pone.0347117 (PMC13089716; doi:10.1371/journal.pone.0347117)
Supplement: S1 Table — (PDF) [file pone.0347117.s001.pdf]

S1 Table. Cross Loading

Items	AAI	AIE	EAIA	KUAI
AA1	**0.928**	0.482	0.671	0.787
AA2	**0.948**	0.441	0.703	0.807
AA3	**0.911**	0.393	0.826	0.716
AE1	0.427	**0.879**	0.398	0.348
AE2	0.439	**0.927**	0.347	0.343
AE3	0.440	**0.941**	0.364	0.392
AE4	0.442	**0.926**	0.355	0.372
AE5	0.367	**0.814**	0.293	0.270
EAA1	0.730	0.332	**0.936**	0.640
EAA2	0.786	0.361	**0.936**	0.690
EAA3	0.733	0.331	**0.946**	0.607
EAA4	0.735	0.404	**0.947**	0.631
EAA5	0.728	0.422	**0.939**	0.596
KUA1	0.712	0.350	0.543	**0.920**
KUA2	0.805	0.375	0.667	**0.955**
KUA3	0.802	0.360	0.671	**0.934**
