# Supplementary material for: AI literacy among basic school teachers in Ghana: A structural equation modelling analysis
Source: PLoS One. 2026 Apr 17;21(4):e0347117. doi: 10.1371/journal.pone.0347117 (PMC13089716; doi:10.1371/journal.pone.0347117)
Supplement: Attachment — Submitted filename: Research report.docx [file pone.0347117.s003.docx]

**Research report**

**Research title**

**Basic School Teachers’ Artificial Intelligence Literacy in Ghana: A Variance-Based Structural Equation Modelling Approach**

**First: Positives of the Research**

**1- Importance of the Topic**

- This study is important because it addresses one of the most prominent requirements of contemporary education: preparing teachers to engage with artificial intelligence (AI) as a fundamental element in developing and enhancing the efficiency of education. Promoting AI literacy among primary school teachers empowers them to understand, apply, and ethically evaluate digital technologies within the classroom, thereby improving the quality of learning and guiding students toward future-oriented skills. The topic is further significant because it addresses a clear knowledge gap related to the limited educational awareness of AI technologies in learning environments, making this research highly valuable both scientifically and practically.

**2- Diversity of Data Collection Tools**

- The study reveals a clear limitation in the diversity of its data collection tools, relying entirely on a single instrument: a Likert-based questionnaire. While questionnaires are suitable for large-scale quantitative research, relying solely on them reduces data richness and limits the ability to interpret phenomena in depth, especially in a complex topic like AI literacy. The absence of complementary tools—such as semi-structured interviews, focus groups, or classroom observations—deprives the study of qualitative data that could explain teachers' motivations, contexts, and actual experiences. It also limits the identification of individual differences and practical challenges that quantitative data alone cannot reveal. Therefore, the limited variety of data collection tools represents a methodological shortcoming that could have been improved in this research.

**3- Integrating Theory with Practice**

- The research demonstrates a strong ability to bridge theory and practice. It begins with a clear theoretical framework for AI literacy, encompassing its four dimensions, and then applies this framework practically in hypothesis building, tool design, and variable analysis. It also offers a direct application of these concepts through structural equation modeling to examine how knowledge, application, assessment, and ethics are reflected in teachers' practices. However, the integration of theory and practice needs further strengthening by including real-world examples or case studies that demonstrate how these dimensions manifest in everyday educational contexts. This would provide a deeper understanding of how theoretical concepts translate into actual professional behavior within classrooms.

**4- Educational and Affective Impact**

- The research reflects a clear educational and affective impact by highlighting how enhancing AI literacy among teachers can improve the quality of education and increase the effectiveness of the classroom environment. Educationally, the research demonstrates that teachers' practical and critical knowledge of AI tools enables them to design more effective teaching practices and helps students develop the analytical thinking and digital skills necessary for the future. Affectively, the research shows that teachers' understanding of and positive engagement with AI can reduce their fears about technology and boost their professional confidence and motivation to adopt innovative teaching methods. However, the affective impact still requires further investigation, particularly regarding feelings of anxiety, acceptance, and attitudes toward AI tools, which could provide a more comprehensive view of the effects of this type of knowledge on educational practices.

**5- Clarity of Analysis**

- The analysis in this research is characterized by a high degree of clarity and organization. The researchers employ a precise quantitative methodology supported by structural equation modeling and present the results in a systematic and easily understandable manner. Measurement indicators, validity and reliability results, path coefficients, and statistical interpretation values are presented clearly and directly, helping the reader grasp the relationships between variables without unnecessary complexity. The use of tables and figures in presenting the results further enhances clarity and allows the reader to verify the accuracy of the conclusions. However, despite the clarity of the statistical analysis, the interpretive explanation of the results could have been strengthened and more deeply connected to real-world educational contexts to ensure a smooth transition from quantitative analysis to educational implications. Overall, the clarity of the analysis remains good and enables the reader to easily comprehend the results.

**6 - Discussion of the results**

- The discussion of the research findings provides a clear and coherent interpretation of the statistical data. Researchers logically connect the results to the theoretical framework and previous studies, explaining the reasons for accepting some hypotheses and rejecting others in light of teachers' practices and professional contexts. The discussion offers plausible explanations for unexpected results, highlighting the impact of practical experience and a lack of ethical awareness on shaping AI literacy levels. While international comparisons and broader linking of the results to training challenges could be expanded, the discussion remains concise, clear, and supports a comprehensive understanding of the fundamental relationships between the study variables.

**Second: Points to improve research quality**

**1- Title and Abstract:**

- The quality of the research can be improved by revising the title and abstract to be more precise and engaging. The current title is clear but lengthy and contains technical details that may weaken its impact. It would be better to shorten it and phrase it in a way that highlights the scientific value of the study, such as: “AI Literacy among Basic School Teachers in Ghana: A Structural Equation Modelling Analysis” or “Understanding AI Literacy in Ghana’s Basic Schools: Insights from a Structural Equation Model,” which are more focused and meaningful titles. As for the abstract, while it contains the most important elements of the research, it tends toward general description without adequately highlighting the research problem or the original contribution of the study. It also does not clearly explain how the results contribute to policy development or teacher training. Therefore, it is advisable to revise it to be more concise and in-depth, with a clearer connection between the research gap, the methodology, the results, and the educational implications.

**2- Introduction and Research Gap:**

- The introduction provides a good background on the importance of artificial intelligence in education, but it remains overly descriptive at the expense of focusing on the main research problem, making the transition to the research gap less sharp and clear. It would have been better if the introduction had included a more direct formulation of the research problem, highlighting the shortcomings of previous studies, particularly the scarcity of research exploring AI literacy among primary school teachers. The research gap could also be strengthened by demonstrating what distinguishes this study from previous work in terms of methodology, sample size, or theoretical framework. Revising the introduction to be more concise and insightful, and directly linking it to the lack of scientific evidence in the Ghanaian context, would enhance the study's rationale and underline its scientific relevance.

**3. Theoretical Framework and Hypothesis Development:**

- The research bases its theoretical framework on a clear model of AI literacy with its four dimensions, a suitable choice consistent with the study's objective. However, the theoretical presentation appears lengthy in some places and leans more towards description than critical analysis. The quality could have been enhanced by clarifying the rationale for choosing this specific framework and discussing its strengths and limitations compared to other models found in the literature. While the development of the hypotheses is logical, it requires more precise formulation that directly and clearly links each hypothesis to its supporting theoretical or experimental evidence, while reducing repetition and improving consistency between premises and conclusions. Overall, enhancing the analytical depth, succinctly presenting the arguments, and more closely connecting the literature to the hypotheses would contribute to improving the quality of this section.

**4. Data Collection and Measurement Tools:**

- The research relies on a single data collection tool—a Likert-based questionnaire—which provides suitable quantitative consistency for statistical analysis but limits the diversity of data sources and reduces the ability to interpret phenomena in depth. The quality of the research could have been enhanced by using additional tools such as interviews, focus groups, or classroom observations to obtain qualitative data that contributes to understanding the contexts and actual experiences of teachers. It would also have been beneficial to clarify the tool's suitability to the cultural and educational context and to describe the steps taken to verify face and content validity before its application. Although the study adopted a scale established in previous literature, expanding the measurement tools, justifying their selection, and clarifying the development procedures would have strengthened the reliability and accuracy of the results.

**7. Validity, Reliability, and Discrimination:**

- The research presents a satisfactory analysis of the measurement quality in terms of validity and reliability, reporting high scores for Cronbach's coefficient, composite reliability, and extracted mean variance, reflecting good internal consistency among the items. However, this section could be strengthened by clarifying the procedures for verifying peripheral and hierarchical validity and providing deeper justifications for the selection of indicators used, rather than simply presenting the statistical values. The presentation could also be enhanced by explaining the implications of these values for model quality and their relevance to the study's educational context, as well as offering a critical reading of the levels of discrimination between variables, compared with the results of similar studies. Providing a more explanatory, rather than merely descriptive, analysis would enhance the reader's confidence in the model's validity and highlight the strength of the measurement instruments used.

**8. Statistical Analysis (PLS-SEM and ANN):**

- Statistical analysis using structural equation modeling (PLS-SEM) demonstrates a good level of organization and provides key indicators such as path coefficients, interpretation coefficients, and effect size. However, the presentation remains descriptive in many instances and requires deeper interpretation of the significance, strength, and relevance of the results to the educational context. Furthermore, the analysis lacks the integration of advanced artificial intelligence models such as artificial neural networks (ANNs), which have become increasingly common in recent studies to enhance prediction and support PLS-SEM findings, particularly when dealing with multidimensional data, as in AI-assisted literacy research. Integrating ANNs could have improved the model's robustness, allowed for testing of nonlinear relationships, and enabled more precise identification of the most influential variables. Additionally, the section's quality could be enhanced by discussing the rationale for choosing PLS-SEM over other models, explaining its suitability to the data type and sample size, and providing a concise comparison highlighting its advantages and limitations. Overall, the statistical analysis is procedurally sound, but requires deeper interpretation and the integration of advanced analytical techniques to maximize the scientific value of the results.

**9. Discussion and Contributions:**

- The discussion includes a clear explanation of the results and a satisfactory connection to the literature, but it tends to be more descriptive than analytical, thus lacking depth. It could be improved by clarifying the implications of the results in an educational context, highlighting the study's actual scientific contribution more clearly, and offering more specific and practical recommendations. Overall, the section is good but needs greater focus and depth to strengthen the research's scientific and practical value.

**10. Ethical considerations:**

- The research primarily addresses ethical considerations by mentioning the acquisition of formal consent, ensuring participant confidentiality, and securing their informed consent, reflecting an acceptable commitment to ethical standards. However, this section could be enhanced by providing a more detailed explanation of data protection procedures, mechanisms for maintaining anonymity, and how any potential risks to participants are addressed, as well as clarifying the extent to which these procedures align with international ethical standards in educational research. In short, the section meets the minimum requirements but needs further elaboration to demonstrate stronger ethical rigor.

**11. Presentation, Language, and Formatting:**

- The research presents its content clearly in general, but the quality of the presentation could be improved by addressing certain aspects of language and organization. There are instances of lengthy sentences and repetition of ideas, which detract from the strength of the academic style and make reading less smooth. The consistency of terminology and the standardization of translations for key concepts could be enhanced to ensure scientific accuracy. Furthermore, the research could benefit from improved formatting of tables and figures, as well as a more uniform style of headings and spacing, which would enhance professionalism and make it easier for the reader to follow the ideas. Overall, the presentation is good but requires further editing and stylistic refinement to improve readability and flow.

**12. References**

- The research draws on a wide range of relatively recent references, which enhances its scientific credibility. However, the quality of the references section could be improved by ensuring consistency in documentation style and standardizing citations according to a single style guide (such as APA). It is also advisable to verify the completeness of data in some sources, update older or less relevant references if any, and emphasize reliance on reputable and up-to-date studies in the field of AI literacy and digital education. Overall, the section is good but requires further refinement in formatting and documentation accuracy to ensure completeness and professionalism.

**Final Evaluation:** Acceptance with Major Revisions

**Reason:**

- The research warrants significant revisions because several key aspects require substantial attention before it can be deemed suitable for publication. The introduction fails to clearly highlight the research gap, and the theoretical framework and hypotheses need further refinement and critical engagement with the literature. Additionally, the data collection methodology lacks diversity, and the discussion leans more towards description than in-depth analysis. Furthermore, certain sections—such as the statistical analysis, abstract, title, and bibliography—require significant organizational, linguistic, and methodological improvements. Therefore, the scope of the required revisions extends far beyond simple stylistic adjustments, necessitating a complete overhaul and development of several core components of the research.

**Thank you very much**
