# Supplementary material for: AI literacy among basic school teachers in Ghana: A structural equation modelling analysis
Source: PLoS One. 2026 Apr 17;21(4):e0347117. doi: 10.1371/journal.pone.0347117 (PMC13089716; doi:10.1371/journal.pone.0347117)
Supplement: Attachment — Submitted filename: RESPONSE TO REVIEWERS COMMENTS.docx [file pone.0347117.s005.docx]

**RESPONSE TO REVIEWERS’ COMMENTS**

Manuscript ID: **PONE-D-25-19123**

Topic: **Basic School Teachers’ Artificial Intelligence Literacy in Ghana: A Variance-Based Structural Equation Modelling Approach**

Refined Topic (Based on the Reviewer’s recommendation): **AI Literacy among Basic School Teachers in Ghana: A Structural Equation Modelling Analysis**

Journal: **PLOS One**

Dear Editor,

We would like to thank you and the reviewers for reviewing this manuscript. Please, we have responded to the comments as follows:

**Journal Requirements:**

When submitting your revision, we need you to address these additional requirements.

1. Please ensure that your manuscript meets PLOS ONE's style requirements, including those for file naming. The PLOS ONE style templates can be found at

<https://journals.plos.org/plosone/s/file?id=wjVg/PLOSOne_formatting_sample_main_body.pdf> and

[https://journals.plos.org/plosone/s/file?id=ba62/PLOSOne_formatting_sample_title_authors_affiliations.pdf](https://track.editorialmanager.com/CL0/https:%2F%2Fjournals.plos.org%2Fplosone%2Fs%2Ffile%3Fid=ba62%2FPLOSOne_formatting_sample_title_authors_affiliations.pdf/1/010f019b8bf5c89f-3e942112-531d-487d-a30e-1e1111dfeb9d-000000/vHFHM-LeHnlc3_zCUWFyChlb3SpRk6rNGO4fTomX-eA=244)

Response: Thank you for this comment. Please, we have rectified it as follows:


**AI Literacy among Basic School Teachers in Ghana: A Structural Equation Modelling Analysis**

Valentina Arkorful^1^, Francis Arthur^2*^, Ernest Opoku^3^, Ayishatu Ameen^4^, Iddrisu Salifu^5^, Sharon Abam Nortey^2^, Solomon Adjatey Tetteh^2^

^1^ College of Distance Education, University of Cape Coast, Cape Coast, Ghana

^2^ Department of Business and Social Sciences Education, Faculty of Humanities and Social Sciences Education, University of Cape Coast, Cape Coast, Ghana

^3^ Institute of Education, School of Educational Development and Outreach, College of Education Studies

^4^ Department of Agricultural Economics and Extension, School of Agriculture, University of Cape Coast, Cape Coast, Ghana

^5^ Centre for Coastal Management- Africa Centre of Excellence in Coastal Resilience, Department of Fisheries and Aquatic Sciences, University of Cape Coast, Cape Coast, Ghana

^*^ Corresponding author

E-mail: a.francis1608@gmail.com/ francis.arthur007@stu.ucc.edu.gh (FA)

2. In the online submission form, you indicated that “Data will be made available on reasonable request.”

All PLOS journals now require all data underlying the findings described in their manuscript to be freely available to other researchers, either 1. In a public repository, 2. Within the manuscript itself, or 3. Uploaded as supplementary information.

This policy applies to all data except where public deposition would breach compliance with the protocol approved by your research ethics board. If your data cannot be made publicly available for ethical or legal reasons (e.g., public availability would compromise patient privacy), please explain your reasons on resubmission and your exemption request will be escalated for approval.

Response: Thank you for this comment. Please, we have indicated that:

**Availability of data and materials**

The datasets generated and/or analysed during the current study are not publicly available due to ethical and legal restrictions imposed by the University of Cape Coast (UCC) Institutional Review Board (IRB), which approved the study protocol. The data contain potentially sensitive information from human participants, including responses that could indirectly identify individuals or reveal personal and professional characteristics of basic school teachers. Public deposition of such data would therefore violate the conditions under which ethical approval was granted, as well as participants’ informed consent agreements guaranteeing confidentiality and anonymity.

However, the data can be made available upon reasonable request for academic and non-commercial research purposes, subject to approval and in compliance with applicable ethical guidelines. Requests for data access should be directed to the Institutional Review Board (IRB), University of Cape Coast, via the official contact: [irb@ucc.edu.gh](mailto:irb@ucc.edu.gh). The IRB serves as an independent, non-author body responsible for reviewing and approving data access requests to ensure adherence to ethical standards and participant confidentiality.

3. Your ethics statement should only appear in the Methods section of your manuscript. If your ethics statement is written in any section besides the Methods, please delete it from any other section.

Response: Thank you for this comment. Please, have deleted the section which was captioned “Ethics approval” under the “declarations”.

4. Please ensure that you refer to Figure 3 in your text as, if accepted, production will need this reference to link the reader to the figure.

Response: Thank you for this comment. Please, we have stated that:

Figure 3 shows the bootstrapping results from PLS-SEM.

(see “**Evaluation of Structural Model**” section of the manuscript)

5. We note you have included a table to which you do not refer in the text of your manuscript. Please ensure that you refer to Table 4 in your text; if accepted, production will need this reference to link the reader to the Table.

Response: Thank you for this comment. Please, we have referred to Table 4 in the text. (see “Discriminant validity” section of the manuscript)

6. If the reviewer comments include a recommendation to cite specific previously published works, please review and evaluate these publications to determine whether they are relevant and should be cited. There is no requirement to cite these works unless the editor has indicated otherwise. 

[Note: HTML markup is below. Please do not edit.]

Response: Thank you for this comment.

Reviewers' comments:

Reviewer's Responses to Questions

**Comments to the Author**

1. Is the manuscript technically sound, and do the data support the conclusions?

The manuscript must describe a technically sound piece of scientific research with data that supports the conclusions. Experiments must have been conducted rigorously, with appropriate controls, replication, and sample sizes. The conclusions must be drawn appropriately based on the data presented.

Reviewer #1: Partly

Reviewer #2: Partly

**Response:** Thank you for the comments. Please, the manuscript is technically sound and also, the data support the conclusions of the study.

2. Has the statistical analysis been performed appropriately and rigorously?

Reviewer #1: Yes

Reviewer #2: Yes

**Response:** Thank you for this comment.

3. Have the authors made all data underlying the findings in their manuscript fully available?

The [PLOS Data policy](http://track.editorialmanager.com/CL0/http:%2F%2Fwww.plosone.org%2Fstatic%2Fpolicies.action%23sharing/1/010f019b8bf5c89f-3e942112-531d-487d-a30e-1e1111dfeb9d-000000/pfGeTiSGJNea31rCBHSa_DAgDsBKceBf5RfqH9sF0fA=244) requires authors to make all data underlying the findings described in their manuscript fully available without restriction, with rare exception (please refer to the Data Availability Statement in the manuscript PDF file). The data should be provided as part of the manuscript or its supporting information, or deposited to a public repository. For example, in addition to summary statistics, the data points behind means, medians and variance measures should be available. If there are restrictions on publicly sharing data—e.g. participant privacy or use of data from a third party—those must be specified.

Reviewer #1: No

Reviewer #2: No

**Response:** Dear Reviewer, thank you for the comments. Please, we indicated that:

**Availability of data and materials**

The datasets generated and/or analysed during the current study are not publicly available due to ethical and legal restrictions imposed by the University of Cape Coast (UCC) Institutional Review Board (IRB), which approved the study protocol. The data contain potentially sensitive information from human participants, including responses that could indirectly identify individuals or reveal personal and professional characteristics of basic school teachers. Public deposition of such data would therefore violate the conditions under which ethical approval was granted, as well as participants’ informed consent agreements guaranteeing confidentiality and anonymity.

However, the data can be made available upon reasonable request for academic and non-commercial research purposes, subject to approval and in compliance with applicable ethical guidelines. Requests for data access should be directed to the Institutional Review Board (IRB), University of Cape Coast, via the official contact: [irb@ucc.edu.gh](mailto:irb@ucc.edu.gh). The IRB serves as an independent, non-author body responsible for reviewing and approving data access requests to ensure adherence to ethical standards and participant confidentiality.

4. Is the manuscript presented in an intelligible fashion and written in standard English?

PLOS ONE does not copyedit accepted manuscripts, so the language in submitted articles must be clear, correct, and unambiguous. Any typographical or grammatical errors should be corrected at revision, so please note any specific errors here.

Reviewer #1: Yes

Reviewer #2: No

**Response:** Thank you for the comments. Please, the manuscript has been presented in an intelligible fashion and written in standard English.

5. Review Comments to the Author

Please use the space provided to explain your answers to the questions above. You may also include additional comments for the author, including concerns about dual publication, research ethics, or publication ethics. (Please upload your review as an attachment if it exceeds 20,000 characters)

Reviewer #1: The manuscript presents a relevant study of AI literacy among basic school teachers in Ghana using a robust variance-based SEM approach. The main findings are novel and the statistical analysis is appropriate. However, substantial revisions are needed before publication.

**Response:** Dear Reviewer, thank you for the comments. Please, we have addressed the comments below.

Major comments:

1) Please streamline the introduction and literature review, reducing repetition and focusing on the main research gaps and rationale.

**Response:** Dear Reviewer, thank you for the comments. Please, we have streamline and introduction and literature review. (see “Revised version of the manuscript).

2) Expand on sampling criteria, potential sources of bias (e.g. only teachers aware of AI), and discuss how this may affect generalizability.

**Response:** Dear Reviewer, thank you for the comments. Please, we indicated that:

Teachers were purposively selected based on their prior awareness of AI, which, while ensuring informed responses, may introduce selection bias and limit the generalizability of the findings to the broader population of basic school teachers in Ghana who may have limited or no exposure to AI.

(see “Study design an population” section of the revised manuscript)

3) Clearly present all model limitations, ensuring interpretation of nonsignificant paths and overall study constraints is balanced and cautious.

**Response:** Dear Reviewer, thank you for the comments. Please, we interpreted all the non-significant paths. (see “Evaluation of structural model” section of the revised manuscript).

4) Revise the Data Availability Statement to comply with PLOS ONE policy: data must be fully accessible through a public repository or similar resource.

**Response:** Dear Reviewer, thank you for the comments. Please, we indicated that:

**Availability of data and materials**

The datasets generated and/or analysed during the current study are not publicly available due to ethical and legal restrictions imposed by the University of Cape Coast (UCC) Institutional Review Board (IRB), which approved the study protocol. The data contain potentially sensitive information from human participants, including responses that could indirectly identify individuals or reveal personal and professional characteristics of basic school teachers. Public deposition of such data would therefore violate the conditions under which ethical approval was granted, as well as participants’ informed consent agreements guaranteeing confidentiality and anonymity.

However, the data can be made available upon reasonable request for academic and non-commercial research purposes, subject to approval and in compliance with applicable ethical guidelines. Requests for data access should be directed to the Institutional Review Board (IRB), University of Cape Coast, via the official contact: [irb@ucc.edu.gh](mailto:irb@ucc.edu.gh). The IRB serves as an independent, non-author body responsible for reviewing and approving data access requests to ensure adherence to ethical standards and participant confidentiality.

(see “**Availability of data and materials**” section)

5) Integrate the Ethics, Funding, and Conflict of Interest disclosures appropriately within the manuscript sections.

**Response:** Dear Reviewer, thank you for the comments. Please, we indicated that:

**Funding**

The authors have confirmed that they did not receive any financial support for the research, authorship, and publication of this article.

**Competing interests**

The authors declare that they have no competing interests.

Also, the Ethics part of the study is at the materials and methods section. This was made so based on the Editor’s recommendation. Thank you

We indicated that:

**Ethical considerations**

The study prioritised the ethical treatment of participants. Ethical approval for the study was granted by the Institutional Review Board of the University of Cape Coast (ID: UCCIRB/CES/2023/169). Following the approval, data collection took place from March 04, 2024, to July 26, 2024. All participants were made to sign written informed consent before taking part, and the researchers guaranteed the confidentiality and anonymity of their responses. In accordance with ethical guidelines, participants were free to withdraw from the study at any time without consequence. To ensure data security and privacy, information was stored securely and only the research team had access to it.

Minor comments:

1) Clarify some ambiguous sentences, especially in the discussion.

**Response:** Dear Reviewer, thank you for the comments. Please, we have rectified all ambiguous sentences, especially in the discussion.

2) Check consistency of terminology and acronyms.

**Response:** Dear Reviewer, thank you for the comments. Please, we have checked and rectified any inconsistency issues in terms of terminology and acronyms.

3) Improve structure: aim for concise paragraphs and clear transitions.

**Response:** Dear Reviewer, thank you for the comments. Please, we have improved the structure of the manuscript.

4) Ensure references are formatted according to PLOS ONE guidelines.

**Response:** Dear Reviewer, thank you for the comments. Please, we have formatted the references to be in line with PLOS ONE guidelines.

Overall, the work is scientifically interesting and potentially suitable for publication after major revision addressing the above points.

**Response:** Dear Reviewer, thank you for the comments. Please, we revised the manuscript based on the suggestions. Thank you

Reviewer #2: The manuscript addresses an important and timely topic concerning AI literacy among basic school teachers, and the study employs an appropriate methodological approach using PLS-SEM. However, several substantial revisions are required to enhance the scientific rigor and clarity of the work.

First, although the statistical analysis is generally appropriate, it would benefit from deeper interpretation and additional analytical validation to strengthen the robustness of the findings.

**Response:** Dear Reviewer, thank you for the comments. Please, the statistical analysis has been well interpreted and stated. (see “Results” section of the manuscript, thank you)

Second, the manuscript does not fully comply with the PLOS data availability policy, as the data are not openly accessible and are instead available only upon request, which is insufficient for publication.

**Response:** Dear Reviewer, thank you for the comments. Please, we have indicated that:


**Availability of data and materials**

The datasets generated and/or analysed during the current study are not publicly available due to ethical and legal restrictions imposed by the University of Cape Coast (UCC) Institutional Review Board (IRB), which approved the study protocol. The data contain potentially sensitive information from human participants, including responses that could indirectly identify individuals or reveal personal and professional characteristics of basic school teachers. Public deposition of such data would therefore violate the conditions under which ethical approval was granted, as well as participants’ informed consent agreements guaranteeing confidentiality and anonymity.

However, the data can be made available upon reasonable request for academic and non-commercial research purposes, subject to approval and in compliance with applicable ethical guidelines. Requests for data access should be directed to the Institutional Review Board (IRB), University of Cape Coast, via the official contact: [irb@ucc.edu.gh](mailto:irb@ucc.edu.gh). The IRB serves as an independent, non-author body responsible for reviewing and approving data access requests to ensure adherence to ethical standards and participant confidentiality.

(see “**Availability of data and materials**” section)

Third, while the paper is understandable, the writing requires considerable language editing to correct grammatical inconsistencies, reduce repetition, and improve the overall clarity and flow of ideas.

**Response:** Dear Reviewer, thank you for the comments. Please, we have done language editing and corrected all grammatical inconsistencies and reduced repetition.

Additionally, several core sections—such as the introduction, theoretical framework, discussion, and methodological justification—would benefit from tighter organization, stronger linkage to prior literature, and clearer articulation of the research gap and contributions.

**Response:** Dear Reviewer, thank you for the comments. Please, we have revised these sections (see “the revised section of the manuscript”)

Ethical considerations are addressed but could be elaborated further to show stronger adherence to international standards. Overall, the manuscript has potential but requires major revisions to address methodological, structural, linguistic, and policy-related issues before it can be considered for publication.

**Response:** Dear Reviewer, thank you for the comments. Please, we have revised the manuscript based on the suggestions.

**RESPONSE TO ATTACHED FILE COMMENTS**

**Research report**

**Research title**

**Basic School Teachers’ Artificial Intelligence Literacy in Ghana: A Variance-Based Structural Equation Modelling Approach**

**First: Positives of the Research**

**1- Importance of the Topic**

- This study is important because it addresses one of the most prominent requirements of contemporary education: preparing teachers to engage with artificial intelligence (AI) as a fundamental element in developing and enhancing the efficiency of education. Promoting AI literacy among primary school teachers empowers them to understand, apply, and ethically evaluate digital technologies within the classroom, thereby improving the quality of learning and guiding students toward future-oriented skills. The topic is further significant because it addresses a clear knowledge gap related to the limited educational awareness of AI technologies in learning environments, making this research highly valuable both scientifically and practically.
- **Response:** Dear Reviewer, thank you for the comments.

**2- Diversity of Data Collection Tools**

- The study reveals a clear limitation in the diversity of its data collection tools, relying entirely on a single instrument: a Likert-based questionnaire. While questionnaires are suitable for large-scale quantitative research, relying solely on them reduces data richness and limits the ability to interpret phenomena in depth, especially in a complex topic like AI literacy. The absence of complementary tools—such as semi-structured interviews, focus groups, or classroom observations—deprives the study of qualitative data that could explain teachers' motivations, contexts, and actual experiences. It also limits the identification of individual differences and practical challenges that quantitative data alone cannot reveal. Therefore, the limited variety of data collection tools represents a methodological shortcoming that could have been improved in this research.

**Response:** Dear Reviewer, thank you for the comments. Please, we have acknowledged it at the limitations section of the manuscript. We indicated that:


Future research could explore alternative methods such as experimental designs and mixed methods approach. Likewise, future studies should focus on the use of advanced AI models such as artificial neural networks (ANNs), which have become increasingly common in recent studies to enhance prediction and support PLS-SEM findings, particularly when dealing with multidimensional data, as in AI-assisted literacy research.

(see “**Strengths and limitation of the study” section of the manuscript)**

**3- Integrating Theory with Practice**

- The research demonstrates a strong ability to bridge theory and practice. It begins with a clear theoretical framework for AI literacy, encompassing its four dimensions, and then applies this framework practically in hypothesis building, tool design, and variable analysis. It also offers a direct application of these concepts through structural equation modeling to examine how knowledge, application, assessment, and ethics are reflected in teachers' practices. However, the integration of theory and practice needs further strengthening by including real-world examples or case studies that demonstrate how these dimensions manifest in everyday educational contexts. This would provide a deeper understanding of how theoretical concepts translate into actual professional behavior within classrooms.

**Response:** Dear Reviewer, thank you for the comments. Please, we have done that (see the revised version of the manuscript)

**4- Educational and Affective Impact**

- The research reflects a clear educational and affective impact by highlighting how enhancing AI literacy among teachers can improve the quality of education and increase the effectiveness of the classroom environment. Educationally, the research demonstrates that teachers' practical and critical knowledge of AI tools enables them to design more effective teaching practices and helps students develop the analytical thinking and digital skills necessary for the future. Affectively, the research shows that teachers' understanding of and positive engagement with AI can reduce their fears about technology and boost their professional confidence and motivation to adopt innovative teaching methods. However, the affective impact still requires further investigation, particularly regarding feelings of anxiety, acceptance, and attitudes toward AI tools, which could provide a more comprehensive view of the effects of this type of knowledge on educational practices.

**Response:** Dear Reviewer, thank you for the comments.

**5- Clarity of Analysis**

- The analysis in this research is characterized by a high degree of clarity and organization. The researchers employ a precise quantitative methodology supported by structural equation modeling and present the results in a systematic and easily understandable manner. Measurement indicators, validity and reliability results, path coefficients, and statistical interpretation values are presented clearly and directly, helping the reader grasp the relationships between variables without unnecessary complexity. The use of tables and figures in presenting the results further enhances clarity and allows the reader to verify the accuracy of the conclusions. However, despite the clarity of the statistical analysis, the interpretive explanation of the results could have been strengthened and more deeply connected to real-world educational contexts to ensure a smooth transition from quantitative analysis to educational implications. Overall, the clarity of the analysis remains good and enables the reader to easily comprehend the results.

**Response:** Dear Reviewer, thank you for the comments. Please, we have done that (see the revised version of the manuscript)

**6 - Discussion of the results**

- The discussion of the research findings provides a clear and coherent interpretation of the statistical data. Researchers logically connect the results to the theoretical framework and previous studies, explaining the reasons for accepting some hypotheses and rejecting others in light of teachers' practices and professional contexts. The discussion offers plausible explanations for unexpected results, highlighting the impact of practical experience and a lack of ethical awareness on shaping AI literacy levels. While international comparisons and broader linking of the results to training challenges could be expanded, the discussion remains concise, clear, and supports a comprehensive understanding of the fundamental relationships between the study variables.

**Response:** Dear Reviewer, thank you for the comments. Please, we

**Second: Points to improve research quality**

**1- Title and Abstract:**

- The quality of the research can be improved by revising the title and abstract to be more precise and engaging. The current title is clear but lengthy and contains technical details that may weaken its impact. It would be better to shorten it and phrase it in a way that highlights the scientific value of the study, such as: “AI Literacy among Basic School Teachers in Ghana: A Structural Equation Modelling Analysis” or “Understanding AI Literacy in Ghana’s Basic Schools: Insights from a Structural Equation Model,” which are more focused and meaningful titles. As for the abstract, while it contains the most important elements of the research, it tends toward general description without adequately highlighting the research problem or the original contribution of the study. It also does not clearly explain how the results contribute to policy development or teacher training. Therefore, it is advisable to revise it to be more concise and in-depth, with a clearer connection between the research gap, the methodology, the results, and the educational implications.

**RESPONSE 1:** Dear Reviewer, thank you for the comments. Please, we have changed based on your suggestion. We indicated it as follows:

**AI Literacy among Basic School Teachers in Ghana: A Structural Equation Modelling Analysis**

**2- Introduction and Research Gap:**

- The introduction provides a good background on the importance of artificial intelligence in education, but it remains overly descriptive at the expense of focusing on the main research problem, making the transition to the research gap less sharp and clear. It would have been better if the introduction had included a more direct formulation of the research problem, highlighting the shortcomings of previous studies, particularly the scarcity of research exploring AI literacy among primary school teachers. The research gap could also be strengthened by demonstrating what distinguishes this study from previous work in terms of methodology, sample size, or theoretical framework. Revising the introduction to be more concise and insightful, and directly linking it to the lack of scientific evidence in the Ghanaian context, would enhance the study's rationale and underline its scientific relevance.

**RESPONSE 2:** Dear Reviewer, thank you for the comments and suggestions. Please, we have revised it as follows:

**Introduction**

The rapid advancement of artificial intelligence (AI) is transforming multiple sectors globally, with education emerging as a key domain of application. AI technologies are increasingly reshaping teaching and learning processes through personalised instruction, automation of administrative tasks, and enhanced student engagement (Chassignol et al., 2018; Gallardo-Montes et al., 2022; Long & Magerko, 2020; Xu et al., 2021). As a result, education systems are under growing pressure to integrate AI meaningfully across all levels, including basic education.

While AI tools such as ChatGPT and Teacherbot offer opportunities to support instructional delivery and improve learning outcomes, their effective use depends largely on teachers’ competencies (Al-Khresheh, 2024; Ding et al., 2024; Pillai et al., 2023). In this context, AI literacy has become a critical requirement for educators. AI literacy encompasses not only knowledge and understanding of AI but also the ability to apply, evaluate, and ethically engage with AI technologies (Ng et al., 2021a; Zhao et al., 2022). Without these competencies, the integration of AI into teaching may be ineffective or even problematic, particularly in relation to issues such as misinformation and ethical misuse (Gaube et al., 2021; Winter et al., 2021).

Despite the increasing relevance of AI in education, evidence suggests that many teachers lack sufficient understanding of AI concepts and their pedagogical implications (Sanusi et al., 2021; Su et al., 2023). This challenge is more pronounced in developing contexts, where technological adoption is growing but often unsupported by adequate capacity building. In Ghana, although AI adoption in education is gradually expanding (Baidoo-Anu & Ansah, 2023), limited attention has been given to assessing teachers’ preparedness, particularly at the basic school level.

Existing studies in Ghana have largely focused on the adoption and use of AI tools, especially generative AI (Essel et al., 2022; Nyaaba & Zhaı, 2024; Nyaaba et al., 2024). However, these studies provide limited insight into teachers’ comprehensive AI literacy and are not specifically centred on basic school teachers. Furthermore, prior research has not consistently applied a structured theoretical framework that captures the multidimensional nature of AI literacy, such as the model proposed by Ng et al. (2021a), which integrates knowledge, application, evaluation, and ethics.

This gap highlights two key limitations in the literature: (1) the lack of empirical evidence on AI literacy among basic school teachers in Ghana, and (2) the absence of studies employing a comprehensive, theory-driven model to examine the interrelationships among AI literacy dimensions. Addressing these limitations is essential for informing policy, curriculum development, and teacher professional training.

Therefore, this study investigates AI literacy among basic school teachers in Ghana using the four-dimensional framework of Ng et al. (2021a) - knowledge and understanding of AI (KUAI), application of AI (AAI), evaluation of AI applications (EAI), and AI ethics (AIE). Unlike previous studies, this research adopts a Variance-Based Structural Equation Modelling (VB-SEM) approach to examine the structural relationships among these dimensions. By doing so, the study provides a more rigorous and comprehensive understanding of AI literacy and contributes both theoretically and empirically to the field.

By fostering BST AI literacy and its implications for classroom practice, this research endeavours to empower teachers to navigate the AI-driven educational landscape effectively. This has long term impact, ultimately enhancing student learning outcomes and preparing the next generation for the challenges and opportunities of the digital age. Thus, this research has the potential to spur innovation and progress within the Ghanaian education sector by providing teachers with AI literacy. The rest of the paper is structured as follows: Section Two presents the hypotheses, while Section Three outlines the research methodology. Section Four presents the results, and Section Five is for the discussion. Finally, Section Six concludes the paper by emphasizing its theoretical significance, practical implications, limitations, and future prospects.

(see “Introduction” section of the revised manuscript, thank you)

**3. Theoretical Framework and Hypothesis Development:**

- The research bases its theoretical framework on a clear model of AI literacy with its four dimensions, a suitable choice consistent with the study's objective. However, the theoretical presentation appears lengthy in some places and leans more towards description than critical analysis. The quality could have been enhanced by clarifying the rationale for choosing this specific framework and discussing its strengths and limitations compared to other models found in the literature. While the development of the hypotheses is logical, it requires more precise formulation that directly and clearly links each hypothesis to its supporting theoretical or experimental evidence, while reducing repetition and improving consistency between premises and conclusions. Overall, enhancing the analytical depth, succinctly presenting the arguments, and more closely connecting the literature to the hypotheses would contribute to improving the quality of this section.

**RESPONSE 3:** Dear Reviewer, thank you for the comments. Please, we have revised the theoretical framework and hypotheses development section as follows:


**Theoretical Framework and Hypotheses Development**

This study adopts the multidimensional conceptualisation of AI literacy proposed by Ng et al. (2021a, 2021b), which extends beyond technical competence to include application, evaluation, and ethical engagement. Within this framework, AI literacy comprises four interrelated dimensions: knowledge and understanding of AI (KUAI), application of AI (AAI), evaluation of AI applications (EAI), and AI ethics (AIE). Although prior studies have defined AI literacy from different perspectives, there is convergence on its integrative nature, combining cognitive, practical, and ethical competencies (Long & Magerko, 2020; Ng et al., 2021a; Zhao et al., 2022). Building on this foundation, the study examines the structural relationships among these dimensions in the context of BST.

***Knowledge and understanding of AI and evaluation of AI application***

Knowledge and understanding of AI (KUAI) constitute the foundation of AI literacy and shape teachers’ ability to critically evaluate AI tools. Empirical evidence indicates that teachers with stronger AI knowledge demonstrate higher professional competence and more effective instructional practices (Zhao et al., 2022). Conceptually, AI literacy involves the ability to interpret and critically engage with AI systems (Long & Magerko, 2020), while also incorporating evaluative and ethical judgement (Ng et al., 2021a). Synthesising these perspectives suggests that evaluation is inherently knowledge-driven. Without adequate understanding, BST are less able to assess the reliability, limitations, and pedagogical value of AI applications. Conversely, stronger KUAI enhances critical judgement and informed decision-making in classroom use. Therefore, we hypothesised that:

**H1:** “*Knowledge and understanding of AI has a significant positive influence on the evaluation of AI applications”.*

***Application of AI and knowledge and understanding of AI***

The relationship between AI application (AAI) and knowledge is dynamic and reciprocal. Practical engagement with AI tools exposes teachers to real-world functionalities, thereby strengthening their conceptual understanding (Zhao et al., 2022). Evidence suggests that effective AI integration depends on teachers’ ability to connect theoretical knowledge with practical use, particularly in designing meaningful learning experiences (Lin & Van Brummelen, 2021). Through continued use, teachers refine their understanding of AI systems, including their capabilities and limitations. Thus, application serves as a mechanism for deepening knowledge and understanding. Therefore, we hypothesised that:

**H2a:** “*Application of AI has a significant positive influence on knowledge and understanding of AI”.*

***Application of AI and AI ethics***

The application of AI in education introduces ethical concerns related to bias, transparency, and responsible use (Jobin et al., 2019; Qin et al., 2020; Xu, 2020). As teachers engage with AI tools, they become more aware of these issues and develop the capacity to apply ethical principles in practice. Ethical considerations are therefore embedded within the application process, guiding how AI is used in instructional contexts (Borenstein & Howard, 2021; Du et al., 2024). Empirical evidence further suggests that hands-on experience with AI enhances teachers’ ability to recognise and address ethical implications (Holmes et al., 2022; Zhao et al., 2022). Therefore, the researchers hypothesised that:

**H2b:** “*AI application has a significant positive effect on AI ethics”.*

***Application of AI and evaluation of AI application***

AI application extends beyond operational use to include critical assessment of AI tools. Teachers who actively engage with AI are better positioned to evaluate its effectiveness, limitations, and impact on learning outcomes (Ahmad et al., 2022; Zhao et al., 2022). Practical experience facilitates informed judgement, enabling teachers to assess alignment with pedagogical goals and ethical standards. This suggests that application strengthens evaluative capacity by providing experiential knowledge of AI systems. Therefore, we proposed the following hypothesis:

H2c: “*The application of AI has a significant positive influence on evaluation of AI”.*

***AI ethics and evaluation of AI application***

AI ethics (AIE) plays a critical role in shaping how teachers evaluate AI technologies. Ethical awareness enables teachers to assess AI tools in terms of fairness, accountability, and transparency, which are essential for responsible classroom integration (Long & Magerko, 2020; Ng et al., 2023). Without ethical grounding, evaluation may overlook critical risks such as bias or misuse. Empirical studies indicate that ethical competence enhances teachers’ ability to make informed and responsible decisions regarding AI adoption (Zhao et al., 2022). Hence, we postulated that:

H3_a_: *“AI ethics has a significant positive influence on the evaluation of AI application”.*

**AI ethics and knowledge and understanding of AI**

AI ethics contributes to deeper understanding by encouraging reflective engagement with AI technologies. Ethical considerations prompt teachers to examine how AI systems function and their broader societal implications, thereby strengthening conceptual knowledge (Ng et al., 2021a; Zhao et al., 2022). This relationship highlights the integrative nature of AI literacy, where ethical awareness supports cognitive development. Teachers with stronger ethical understanding are therefore more likely to develop a comprehensive grasp of AI concepts and their applications. Consequently, this study also hypothesised that:

H_3b_: *“AI ethics positively influences knowledge and understanding of AI”*.

(see this section in the revised manuscript, thank you)

**4. Data Collection and Measurement Tools:**

- The research relies on a single data collection tool—a Likert-based questionnaire—which provides suitable quantitative consistency for statistical analysis but limits the diversity of data sources and reduces the ability to interpret phenomena in depth. The quality of the research could have been enhanced by using additional tools such as interviews, focus groups, or classroom observations to obtain qualitative data that contributes to understanding the contexts and actual experiences of teachers. It would also have been beneficial to clarify the tool's suitability to the cultural and educational context and to describe the steps taken to verify face and content validity before its application. Although the study adopted a scale established in previous literature, expanding the measurement tools, justifying their selection, and clarifying the development procedures would have strengthened the reliability and accuracy of the results.

**RESPONSE 4:** Dear Reviewer, thank you for the comments. Please, we have indicated that future studies should be longitudinal in nature and also, use experimental designs and mixed methods approach (see “**Strengths and limitation of the study**” section of the manuscript)

Also, the reliability of the items have been included in the manuscript. We indicated that:

**Measurement model assessment**

To make sure the assessment tool is reliable and accurate, we checked how consistent and valid the variables are. Table 1 shows the detailed results for these checks, including factor loadings, different measures of reliability, and measures of variance for each construct: Cronbach's alpha (α), Rho_A, composite reliability (CR), average variance extracted (AVE), and inner variance inflation factor (VIF)

**Indicator loadings and construct reliability**

All items had factor loadings above the threshold of 0.70, signifying that each item contributed significantly to its respective construct. The loadings ranged from 0.814 to 0.948 across the constructs (see Table 1), indicating strong correlations between items and their underlying factors. Also, reliability of the constructs was assessed using Cronbach’s alpha (α), composite reliability (CR), and Rho_A. All constructs demonstrated high reliability, with Cronbach’s alpha values well above the acceptable threshold of 0.70, indicating consistent internal reliability. Specifically, the values for Cronbach’s alpha were 0.921 for Applying AI (AAI), 0.940 for AI Ethics (AIE), 0.968 for Evaluating AI Application (EAIA), and 0.930 for Knowing and Understanding AI (KUAI) [see Table 1].

Additionally, the composite reliability (CR) values for all constructs exceeded the recommended threshold of 0.70, confirming the internal consistency of the constructs (Hair et al., 2022). The CR values were 0.950 for AAI, 0.954 for AIE, 0.975 for EAIA, and 0.955 for KUAI. Additionally, Rho_A values, which provide an alternative measure of reliability, were also high, corroborating the reliability of the constructs.

**Convergent validity**

Construct validity was assessed through average variance extracted (AVE). The AVE values for each construct were well above the minimum acceptable level of 0.50, which indicates a good level of convergent validity (Hair *et al*., 2022). The AVE values were 0.864 for AAI, 0.808 for AIE, 0.886 for EAIA, and 0.876 for KUAI (see Table 1). These high AVE values suggest that the constructs explain a significant portion of the variance in their respective items. The results of the PLS-SEM algorithm are displayed by Figure 2.

**Table 1: Construct Reliability and Validity**

| Constructs | Items | Loadings | α | Rho_A | CR | AVE | p value |
| --- | --- | --- | --- | --- | --- | --- | --- |
| AAI | AA1 | 0.928 | 0.921 | 0.921 | 0.950 | 0.864 | *** |
|  | AA2 | 0.948 |  |  |  |  | *** |
|  | AA3 | 0.911 |  |  |  |  | *** |
| AIE | AE1 | 0.879 | 0.940 | 0.946 | 0.954 | 0.808 | *** |
|  | AE2 | 0.927 |  |  |  |  | *** |
|  | AE3 | 0.941 |  |  |  |  | *** |
|  | AE4 | 0.926 |  |  |  |  | *** |
|  | AE5 | 0.814 |  |  |  |  | *** |
| EAIA | EAA1 | 0.936 | 0.968 | 0.968 | 0.975 | 0.886 | *** |
|  | EAA2 | 0.936 |  |  |  |  | *** |
|  | EAA3 | 0.946 |  |  |  |  | *** |
|  | EAA4 | 0.947 |  |  |  |  | *** |
|  | EAA5 | 0.939 |  |  |  |  | *** |
| KUAI | KUA1 | 0.920 | 0.930 | 0.936 | 0.955 | 0.876 | *** |
|  | KUA2 | 0.955 |  |  |  |  | *** |
|  | KUA3 | 0.934 |  |  |  |  | *** |

Note: AAI = Application of AI; AIE = AI ethics; EAIA = Evaluation of AI application; KUAI = Knowledge and understanding of AI; α = Cronbach’s Alpha; CR = Composite Reliability; AVE = Average Variance Extracted.

(Please, see “Results” section of the manuscript, thank you)

**5. Validity, Reliability, and Discrimination:**

- The research presents a satisfactory analysis of the measurement quality in terms of validity and reliability, reporting high scores for Cronbach's coefficient, composite reliability, and extracted mean variance, reflecting good internal consistency among the items. However, this section could be strengthened by clarifying the procedures for verifying peripheral and hierarchical validity and providing deeper justifications for the selection of indicators used, rather than simply presenting the statistical values. The presentation could also be enhanced by explaining the implications of these values for model quality and their relevance to the study's educational context, as well as offering a critical reading of the levels of discrimination between variables, compared with the results of similar studies. Providing a more explanatory, rather than merely descriptive, analysis would enhance the reader's confidence in the model's validity and highlight the strength of the measurement instruments used.

**RESPONSE 5:** Dear Reviewer, thank you for the comments. Please, the research presents a satisfactory analysis of the measurement quality in terms of validity and reliability, reporting high scores for Cronbach's coefficient, composite reliability, and extracted mean variance, reflecting good internal consistency among the items. It must be noted that it follows the procedure of conducting a structural equation modelling analysis.

(Please, see “Measurement model assessment, indicator loadings and construct reliability, and discriminant validity” sections of the manuscript, thank you)

**6. Statistical Analysis (PLS-SEM and ANN):**

- Statistical analysis using structural equation modeling (PLS-SEM) demonstrates a good level of organization and provides key indicators such as path coefficients, interpretation coefficients, and effect size. However, the presentation remains descriptive in many instances and requires deeper interpretation of the significance, strength, and relevance of the results to the educational context. Furthermore, the analysis lacks the integration of advanced artificial intelligence models such as artificial neural networks (ANNs), which have become increasingly common in recent studies to enhance prediction and support PLS-SEM findings, particularly when dealing with multidimensional data, as in AI-assisted literacy research. Integrating ANNs could have improved the model's robustness, allowed for testing of nonlinear relationships, and enabled more precise identification of the most influential variables. Additionally, the section's quality could be enhanced by discussing the rationale for choosing PLS-SEM over other models, explaining its suitability to the data type and sample size, and providing a concise comparison highlighting its advantages and limitations. Overall, the statistical analysis is procedurally sound, but requires deeper interpretation and the integration of advanced analytical techniques to maximize the scientific value of the results.

**RESPONSE 6:** Dear Reviewer, thank you for the comments. Please, we used structural equation modelling analysis (PLS-SEM) demonstrates a good level of organization and provides key indicators such as path coefficients, interpretation coefficients, and effect size. We thank you for the suggestion of using ANN, however, in this study, we did not use ANN based on the analytical approach of the study.

We have indicated that future studies should focus on:
“the use of advanced artificial intelligence models such as artificial neural networks (ANNs), which have become increasingly common in recent studies to enhance prediction and support PLS-SEM findings, particularly when dealing with multidimensional data, as in AI-assisted literacy research”.

It was stated as follows:

Likewise, future studies should focus on the use of advanced AI models such as artificial neural networks (ANNs), which have become increasingly common in recent studies to enhance prediction and support PLS-SEM findings, particularly when dealing with multidimensional data, as in AI-assisted literacy research.

(Please, see “**Strengths and limitation of the study”** section of the manuscript, thank you)

Thank you

**7. Discussion and Contributions:**

- The discussion includes a clear explanation of the results and a satisfactory connection to the literature, but it tends to be more descriptive than analytical, thus lacking depth. It could be improved by clarifying the implications of the results in an educational context, highlighting the study's actual scientific contribution more clearly, and offering more specific and practical recommendations. Overall, the section is good but needs greater focus and depth to strengthen the research's scientific and practical value.

**RESPONSE 7:** Dear Reviewer, thank you for the comments. Please, we have rectified all issues with the discussion and contributions of the study. We have refined it as follows:

**Discussion**

The use of transformative technologies has become a fundamental aspect of professional development at various sectors and the education sector is no exception. The transformative potential of AI for shaping teaching and learning is undeniable. As AI advances at a rapid pace, it is crucial to understand the multifaceted nature of AI literacy among teachers. Inspired by this, we explored the core dimension of AI literacy, including knowledge and understanding of AI (KUAI), applications of AI (AAI), evaluation of AI applications (EAIA), and ethics of AI (AIE), needed for responsible implementation by primary school teachers. This study used 319 basic school teachers and employed variance-based structural equation modelling (VB-SEM) to test the relationships between the dimensions of AI literacy. The aim of the study was to identify the factors that influence basic school teachers' AI literacy. A total of six hypotheses were tested, of which the results validated three hypotheses and did not support three.

The results of the study showed no significant influence of KUAI on EAIA, suggesting that knowledge and understanding of AI does not significantly influence the evaluation of AI by basic school teachers. This may be because most of these basic school teachers rely on preconceived notions or external opinions, leading to bias or overconfidence. In addition, basic school teachers' theoretical knowledge and understanding of AI may not align with the practical outcomes and real-world effectiveness of AI applications, making their actual AI literacy a less important factor in the evaluation of AI applications. This finding contradicts previous research that emphasises that teachers' knowledge and understanding of AI has closely linked with the evaluation of AI applications (Zhao et al., 2022). Therefore, there is an urgent need to improve the AI literacy of Ghanaian basic school teachers, both in terms of knowledge and understanding of AI (KUAI) and evaluation of AI applications (EAIA). To address this, practical policy implications should include integrating comprehensive AI training programmes into teacher education, providing ongoing professional learning communities (PLCs) focused on AI, and ensuring access to AI resources. This will enable basic school teachers to develop technical knowledge and understanding of AI, leading to the evaluation of AI applications and more informed and effective use of AI in the classroom.

In addition, this study found a significant relationship between AAI and KUAI scores. This highlights that the use of AI by primary school teachers has a significant positive impact on improving their knowledge and understanding of AI, which is a core dimension of AI literacy. This finding is expected and can be attributed to the common notion that practical, hands-on experience in the iterative learning process with increased engagement and motivation are factors that contribute to deepening technological literacy. In this context, basic school teachers can better develop AI literacy in terms of knowledge and understanding to support their teaching practice and improve the overall quality of education if they continue to apply AI tools. The findings of this study are consistent with those of previous research emphasising the importance of teachers' application of AI in determining the knowledge and understanding of AI in their professional practice. Therefore, it is recommended that basic school teachers' knowledge and understanding of AI be further enhanced by encouraging their use of AI tools. This can be achieved by integrating AI-based tools into teacher training, promoting practical AI projects, and providing continuous AI-focused professional development.

This study shows that AI literacy has a significant positive impact on AI ethics among primary school teachers in Ghana. Thus, through the application of AI, teachers develop AI literacy on how to articulate the ethical aspects of AI responsibly in educational settings and share these ideas with their peers and students. This finding supports those of previous studies (Holmes et al., 2022; Zhao et al., 2022) and emphasises the importance of AI literacy. The positive influence of application of AI on AI ethics among Ghanaian primary school teachers can be attributed to increased AI literacy. As teachers become more knowledgeable about AI, they are better equipped to understand ethical considerations and use AI responsibly. This leads to effective and ethical implementation of AI tools in the classroom, promoting a culture of responsible AI use and improving educational outcomes. To ensure that teachers are well prepared to use AI responsibly, it is recommended that AI literacy is integrated into teacher training programmes, that continuous professional development is provided, and that ethical guidelines for the use of AI in education are established.

More importantly, the results showed that AAI has a significant positive impact on EAI. This emphasises that the application of AI by basic school teachers can positively enhance their AI literacy in terms of evaluating the application of AI, which is consistent with the findings of previous studies (Ahmad et al., 2022; Liang, & Han, 2022). Thus, as teachers integrate AI tools into their teaching practice and continuously apply or use them, their understanding and evaluation of AI technology also improves. Teachers' improved AI literacy is likely due to hands-on experience, which enhances their ability to critically evaluate and effectively use AI in educational contexts. Given that the use of AI has a significant positive impact on teachers' AI literacy in terms of evaluating such AI tools, there is a need for policies that promote the integration of AI into teacher training programmes. Basic school teachers should be given hands-on experience with AI to improve their literacy and evaluation skills. Policies should focus on promoting the integration of AI in primary education and provide teachers with continuous training in the use of these technologies. This will enable basic teachers to make effective use of AI, improve their teaching methods and ensure that pupils benefit from advanced technology in the classroom. This policy approach supports sustainable improvements in teaching quality and AI literacy.

The literature suggests that teachers' AI literacy in AI ethics is essential for fostering positive attitudes, knowledge and skills relevant to their future studies and careers. Therefore, one of the questions we wanted to address was whether AI ethics determines teachers' evaluation of AI applications as AI literacy. However, the results showed that AIE had no significant effect on EAI. Thus, our findings suggest that primary school teachers' understanding of AI ethics does not directly affect their ability to evaluate AI applications. While training in AI ethics is crucial, it does not necessarily improve practical evaluation skills. This seems to contradict the findings that knowledge of AI ethics significantly improves the evaluation of AI applications (Ng et al., 2022a; Ng et al., 2023; Zhao et al., 2022). To address this gap, it is therefore necessary to integrate targeted technical training alongside AI ethics in professional development programmes. We recommended that policymakers emphasize hands-on experience with AI tools to improve practical evaluation skills. This would enable primary school teachers to balance knowledge of AI ethics with practical evaluation of applications to ensure comprehensive AI literacy, thereby improving their overall effectiveness in using AI in education.

Furthermore, the literature on AI literacy suggests that AI ethics and knowledge and understanding of AI go hand in hand. However, the results of our study showed that AIE did not significantly influence KUAI. Thus, AI ethics, conceptualised as primary teachers' understanding of the responsible use of AI, does not have a significant effect on knowledge and understanding of AI applications. This finding implies that ethics training alone does not improve teachers' knowledge and understanding of AI applications. However, this contradicts the finding that teachers' AI ethical literacy in turn enhances their knowledge and understanding of AI (Shih et al., 2021; Zhao et al., 2022). To improve teachers' knowledge and understanding of AI, integrate practical AI training with ethics education. Provide hands-on workshops and technical courses focused on AI applications. This combined approach will ensure that teachers gain both ethical insights and practical skills, leading to a well-rounded AI literacy that is essential for effective teaching.

(Please, see “Discussion” and “Contribution and Implications” sections of the manuscript, thank you)

**8. Ethical considerations:**

- The research primarily addresses ethical considerations by mentioning the acquisition of formal consent, ensuring participant confidentiality, and securing their informed consent, reflecting an acceptable commitment to ethical standards. However, this section could be enhanced by providing a more detailed explanation of data protection procedures, mechanisms for maintaining anonymity, and how any potential risks to participants are addressed, as well as clarifying the extent to which these procedures align with international ethical standards in educational research. In short, the section meets the minimum requirements but needs further elaboration to demonstrate stronger ethical rigor.

**RESPONSE 8:** Dear Reviewer, thank you for the comments. Please, we have rectified all issues concerning ethical considerations by mentioning the acquisition of formal consent, ensuring participant confidentiality, and securing their informed consent.
We indicated that:

**Ethical considerations**

The study prioritised the ethical treatment of participants. Ethical approval for the study was granted by the Institutional Review Board of the University of Cape Coast (ID: UCCIRB/CES/2023/169). Following the approval, data collection took place from March 04, 2024, to July 26, 2024. All participants were made to sign written informed consent before taking part, and the researchers guaranteed the confidentiality and anonymity of their responses. In accordance with ethical guidelines, participants were free to withdraw from the study at any time without consequence. To ensure data security and privacy, information was stored securely and only the research team had access to it.

(Please, see “Ethical considerations” section of the manuscript, thank you)

**9. Presentation, Language, and Formatting:**

- The research presents its content clearly in general, but the quality of the presentation could be improved by addressing certain aspects of language and organization. There are instances of lengthy sentences and repetition of ideas, which detract from the strength of the academic style and make reading less smooth. The consistency of terminology and the standardization of translations for key concepts could be enhanced to ensure scientific accuracy. Furthermore, the research could benefit from improved formatting of tables and figures, as well as a more uniform style of headings and spacing, which would enhance professionalism and make it easier for the reader to follow the ideas. Overall, the presentation is good but requires further editing and stylistic refinement to improve readability and flow.

**RESPONSE 9:** Dear Reviewer, thank you for the comments. Please, we have rectified all issues with presentation, language and formatting.

**10. References**

- The research draws on a wide range of relatively recent references, which enhances its scientific credibility. However, the quality of the references section could be improved by ensuring consistency in documentation style and standardizing citations according to a single style guide (such as APA). It is also advisable to verify the completeness of data in some sources, update older or less relevant references if any, and emphasize reliance on reputable and up-to-date studies in the field of AI literacy and digital education. Overall, the section is good but requires further refinement in formatting and documentation accuracy to ensure completeness and professionalism.

**RESPONSE 10:** Dear Reviewer, thank you for the comments. Please, all the references have been formatted to be in line with the APA referencing style.

(Please, see “**References”** section of the manuscript, thank you)
